# A New Murine Model of Chronic Kidney Disease-Mineral and Bone Disorder

**DOI:** 10.1155/2017/1659071

**Published:** 2017-12-14

**Authors:** Bianca Frauscher, Katharina Artinger, Alexander H. Kirsch, Ida Aringer, Foteini Moschovaki-Filippidou, Máté Kétszeri, Corinna Schabhüttl, Peter P. Rainer, Albrecht Schmidt, Tatjana Stojakovic, Astrid Fahrleitner-Pammer, Alexander R. Rosenkranz, Philipp Eller, Kathrin Eller

**Affiliations:** ^1^Clinical Division of Nephrology, Medical University of Graz, Graz, Austria; ^2^Clinical Division of Cardiology, Medical University of Graz, Graz, Austria; ^3^Clinical Institute of Medical and Chemical Laboratory Diagnostics, Medical University of Graz, Graz, Austria; ^4^Clinical Division of Endocrinology and Diabetes, Medical University of Graz, Graz, Austria; ^5^Intensive Care Unit, Department of Internal Medicine, Medical University of Graz, Graz, Austria

## Abstract

Chronic kidney disease (CKD) is associated with mineral and bone disorder (MBD), which is the main cause of the extensively increased cardiovascular mortality in the CKD population. We now aimed to establish a new murine experimental CKD-MBD model. Dilute brown non-Agouti (DBA/2) mice were fed with high-phosphate diet for 4 (HPD4) or 7 (HPD7) days, then with standard chow diet (SCD) and subsequently followed until day 84. They were compared to DBA/2 mice maintained on SCD during the whole study period. Both 4 and 7 days HPD-fed mice developed phosphate nephropathy with tubular atrophy, interstitial fibrosis, decreased glomerular filtration rate, and increased serum urea levels. The abdominal aorta of HPD-treated mice showed signs of media calcification. Histomorphometric analysis of HPD-treated mice showed decreased bone volume/tissue volume, low mineral apposition rate, and low bone formation rate as compared to SCD-fed mice, despite increased parathyroid hormone levels. Overall, the observed phenotype was more pronounced in the HPD7 group. In summary, we established a new, noninvasive, and therefore easy to perform reproducible CKD-MBD model, which showed media calcification, secondary hyperparathyroidism, and low-turnover bone disease.

## 1. Introduction

Chronic kidney disease (CKD) is a major health burden as per the 2011 US Renal Data System Annual Data Report; 15.1% of the US adult population has CKD [1]. CKD per se, but especially end-stage renal disease (ESRD), is associated with high morbidity and mortality [2]. Cardiovascular disease is the single greatest cause of mortality in CKD/ESRD and to a large extent is attributable to abnormal mineral metabolism leading to extensive arterial calcifications, a reduced vascular compliance, left ventricular hypertrophy, and sudden cardiac death [3]. When starting dialysis, 50% of the patients have fractures and the survival rate following a hip fracture is 0% [4, 5].

As opposed to nonuraemic subjects where arterial calcification typically affects intimal atherosclerotic plaques, patients with CKD predominantly develop calcification of the tunica media [6, 7]. It is current knowledge that deregulations in mineral and bone metabolism accompanying CKD are the major driving force for the occurrence of media calcification, which led to the term CKD-mineral and bone disorder (MBD) [8, 9]. It additionally includes renal osteodystrophy, which develops in the majority of CKD patients. The spectrum of renal osteodystrophy ranges from low-turnover adynamic bone disease to high-turnover osteitis fibrosa and more than one type can coexist in the same patient [9]. Bone disease develops due to deregulations in PTH, FGF23, sclerostin, and Vitamin D levels, which are deregulated with declining kidney function [10]. All forms of renal osteodystrophy are accompanied by bone loss finally leading to a significantly increased risk of bone fractures including hip fractures in the CKD population [11, 12]. Especially, patients with adynamic bone disease are prone to develop extensive arterial calcification [4, 5]. Unfortunately, currently available biomarkers such as PTH, FGF23, and alkaline phosphatase can only poorly predict the respective form of renal osteodystrophy and are of limited use in guiding therapy. Thus, according to KDIGO guidelines, bone biopsy and bone histomorphometry remain the gold standard in diagnosing renal osteodystrophy. Yet, it is not recommended to routinely perform bone biopsy in ESRD patients [8, 9]. Nevertheless, few randomized controlled trials have evaluated different treatment strategies depending on the type of renal osteodystrophy. Murine CKD-MBD models are clearly needed to test putative therapeutic strategies for the treatment of vascular calcification and/or renal osteodystrophy. Recently, a subtotal nephrectomy/CKD model with uremic osteodystrophy and abnormalities in bone volume and mineralization has been published [13]. Here, we describe a new, noninvasive, and therefore easy to perform reproducible CKD-MBD model with secondary hyperparathyroidism and media calcification. Contrary to the subtotal nephrectomy/CKD model, we found low-turnover bone disease in our mice.

## 2. Material and Methods

### 2.1. Study Design

Female 8-week-old dilute brown non-agouti 2 (DBA/2NCrl, hereafter referred to as DBA/2) mice were obtained from Charles River (Sulzfeld, Germany) and housed in a virus/antibody-free environment in the laboratory animal facility of the Medical University of Graz. These mice are susceptible to ectopic renal calcification and media calcification when exposed to increased oral phosphate loads [14–16]. In order to cause renal damage, these mice were fed standard chow (SCD; *n* = 8) or high-phosphate diet for 4 (HPD4; *n* = 4) or 7 (HPD7; *n* = 4) days with subsequent return to SCD until day 84 after starting HPD diet. The high-phosphate diet (Altromin, Lage, Germany) contained 20.2 g of phosphorus, 9.4 g of calcium, 0.7 g of magnesium, and 500 IU/kg of vitamin D3. The standard chow contained 7.0 g of phosphorous, 10.0 g of calcium, 2.2 g of magnesium, and 1000 IU/kg of vitamin D3.

All animal experiments were approved by the Committee of the Ethics of Animal Experiments of the Austrian Ministry (BMWFW-66.010/0061-WF/V/3b/2016). All experiments were conducted under strict adherence of the law of Austria.

### 2.2. Metabolic Studies

For metabolic studies, blood was obtained at the end of the experiment from anaesthetized mice by retro-orbital bleeding. Serum urea levels were measured with standard laboratory techniques. Serum fibroblast growth factor 23 (FGF23) levels (Immutopics International, San Clemente, CA, USA) and serum parathyroid hormone (Pth) levels (Immutopics International) were quantified using commercially available enzyme-linked immunosorbent assay kits.

### 2.3. Evaluation of Histopathology, Histomorphometry, and Immunopathology

Formalin-fixed renal tissue and aortas were embedded in paraffin and cut into 4 *μ*m sections prior to staining. The extent of media as well as renal calcification was determined histologically using alizarin red technique. Alizarin red staining was performed by incubating rehydrated paraffin sections in 2% Alizarin Red S solution (Sigma-Aldrich, St. Louis, MO, USA). Additionally, picrosirius red staining was performed by incubating rehydrated renal paraffin section in 0.1% Picrosirius Red solution (Sigma-Aldrich).

For the calcein-labelling, the mice were intraperitoneally injected with 20 mg/kg of calcein (Sigma-Aldrich) 7 and 2 days prior to sacrifice. Thereafter, the tibia was fixed in 70% ethanol and embedded in methyl methacrylate and sectioned. For further analysis, toluidine blue staining was used. Dewaxed and hydrated bone sections were immersed in toluidine blue working solution (1% Toluidine Blue O, Sigma-Aldrich; 2.5% sodium carbonate, 70% ethanol) for 5 minutes. Thereafter, sections were washed in dH2O, dehydrated in n-Butyl acetate and cover slipped. Bone histomorphometric parameters were obtained through tissue sections analysed by OsteoMeasure™ Software (OsteoMetrics, Decatur, GA, USA).

OCT-embedded (Sakura Finetek, St. Torrance, CA, USA) frozen tissue sections (4 *μ*m) were cut for immunohistochemical stainings. The three-layer immunoperoxidase technique was used for the detection of infiltrating macrophages and T cells in the renal tissue. Macrophages were stained using a rat anti-mouse mAb (anti-CD68, clone FA-11; Serotec, Oxford, UK). A semiquantitative scoring system for kidney-infiltrating macrophages was performed as follows: 0 = 0–4 cells stained positive, 1 = 5–10 cells, 2 = 10–50 cells, 3 = 50–200 cells, and 4 = > 200 cells stained positive per low-power field.

For the detection of CD4^+^ and CD8^+^ T cells, rat anti-mouse CD4 (clone YTS191.1; Serotec) and CD8-*α* (clone KT15; Serotec) mAb were used. Biotin-conjugated goat anti-rat IgG antibody (Jackson ImmunoResearch Laboratories, West Grove, PA, USA) was used as a secondary antibody, followed by incubation with a streptavidin-biotin complex (Vector Laboratories, Burlingame, CA, USA) and subsequent development with 0.4% 3-amino-9-ethylcarbazole for 5 minutes and counterstaining with Gill's Haematoxylin. T cell quantitation was performed by counting the number of positive cells in six adjacent high power fields of renal cortex and medulla.

### 2.4. Reverse Transcription Real-Time Polymerase Chain Reaction

Murine tissue was stored at −80°C. Total RNA was isolated from the kidneys using TRI Reagent (Sigma-Aldrich) according to a standard protocol. Thereafter, 2 *μ*g of total RNA was reverse transcribed using SuperScript III Transcription Kit (Invitrogen, Carlsbad, CA, USA) and random primers (Invitrogen). *Hprt* gene was served as the housekeeping reference and was assessed using SYBR Green Master Mix (Invitrogen) and by the following primers: forward 5′ GCT TCC TCA GAC CGG TTT TTG C 3′ and reverse 5′ ATC GCT AAT CAC GAC GCT GGG ACT G 3′. For quantification of *Foxp3*, *Gata3*, *Rorc*, *Tnfa*, *Tbx21*, *Ccr2*, and *CCr5*, the gene expression assays Mm00475162_m1, Mm00484683_m1, Mm01261022, Mm00443258_m1, Mm00450960, Mm00438270, and Mm01216171 (Applied Biosystems, Foster City, DA, USA) were used, respectively.

Real-Time PCR was performed in duplicates on a CF96 real-time detection system (Bio-Rad, Vienna, Austria). The data was evaluated using the 2-^ΔΔ^CT method.

### 2.5. Measurement of the Glomerular Filtration Rate (GFR)

GFR was measured by FITC-inulin clearance. FITC-inulin (Sigma-Aldrich; 5% in 0.85% NaCl) was dialyzed for 24 h against 0.85% NaCl. The dialyzed FITC-inulin solution was sterile filtered and injected intravenously (2 *μ*l/g body weight). Three, 5, 7, 10, 15, 56, and 75 minutes after injection, blood was collected from the tail vein. After centrifugation, plasma was diluted 1 : 10 in 0.5 mol/L HEPES and fluorescence was measured. GFR was calculated using a two-compartment model of two-phase exponential decay.

### 2.6. Mouse Echocardiography

2D-guided M-mode echoes (30 MHz) were obtained from short- and long-axis views at the level of the largest LV-diameter using a VS-VEVO 770 High-Resolution Imaging System (Visualsonics, Toronto, Canada) equipped with a 30 MHz RMV (Real-time micro-visualization) scan head. Mice were lightly anesthetized with 2% isoflurane and were allowed to breathe spontaneously. The chest was shaved, acoustic coupling gel was applied, and a warming pad was used to maintain normothermia. Mice were imaged in a shallow left lateral decubitus position. LV end-diastolic (EDD) and end-systolic (ESD) dimensions were measured from original tracings by using the leading edge convention of the American Society of Echocardiography. LV percent fractional shortening (LVFS), LV mass (LVM), and end-diastolic wall-thickness/cavity ratio were calculated as previously described [17].

### 2.7. Statistical Analysis

Results from experiments are shown as means ± SEM. Testing for normal distribution was done using the Kolmogorov-Smirnov test with Dallal-Wilkinson-Lilliefors correction. When comparing the two groups, according to the distribution nonparametric Mann–Whitney *U* test or unpaired Student's *t*-test was used. When comparing the three groups, ANOVA or Kruskal-Wallis test was performed with subsequent Dunn's test with adjustment for multiple comparisons. A two-sided *P* < 0.05 was considered statistically significant. All statistical analyses were performed using GraphPad Prism 6.0 (GraphPad Software, La Jolla, CA, USA).

## 3. Results

### 3.1. High-Phosphate Diet for 4 and 7 Days Induces Chronic Kidney Disease (CKD) after 84 Days Follow-Up

**Figure 1:**
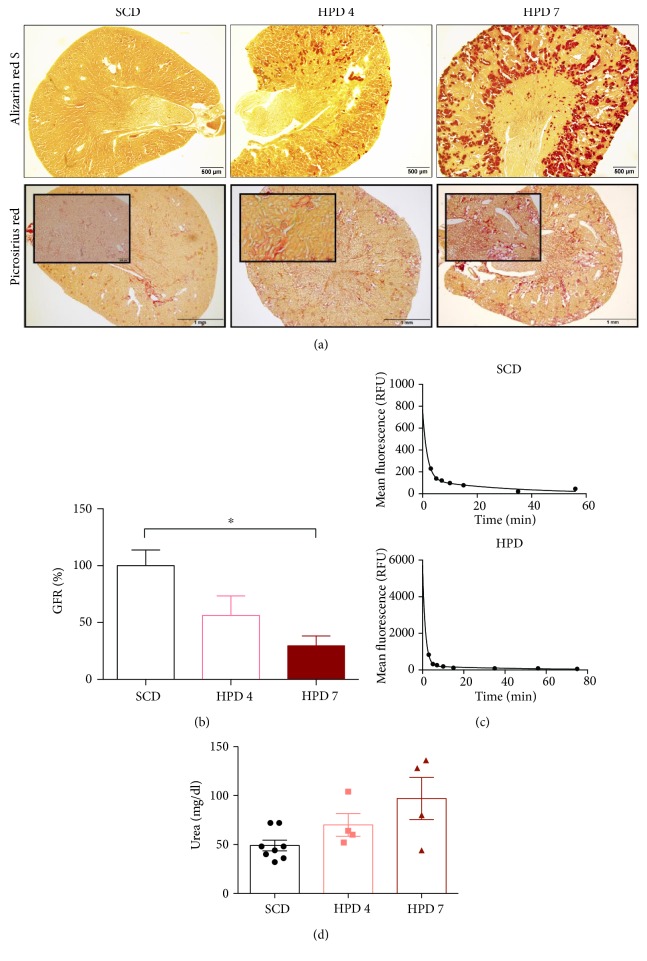
Kidney phenotype of the CKD model. Mice were either fed with HPD for 4 (HPD4, *n* = 4) or 7 days (HPD7, *n* = 4) and then followed on SCD until day 84. They were compared with mice on SCD for the complete study period (SCD, *n* = 8). (a) Kidneys were evaluated for calcium hydroxyapatite crystals and fibrosis by performing alizarin red and sirius red stain, respectively. Representative pictures are provided. (b) On day 84, FITC-inulin clearance to evaluate the glomerular filtration rate (GFR) was performed. The GFR in percentage compared to SCD is provided. (c) Representative GFR curves of SCD and 7-day HPD 7 mice are provided. (d) Serum urea was evaluated in mice on day 84. ^∗^*p* < 0.05.

Female DBA/2 mice were fed for either 4 (HPD4) or 7 (HPD7) days with high-phosphate diet and then followed until day 84 on standard chow (SCD). They were compared to DBA/2 mice on SCD. At day 84, all mice subjected to the HPD developed phosphate nephropathy with tubular calcium hydroxyapatite crystals, which was stained positive with alizarin red. Only small and significantly less alizarin red positive deposits were found in DBA/2 mice on standard chow (SCD) (Figure 1(a), upper panel). To evaluate fibrosis, a sirius red stain was performed. All HPD mice displayed significantly increased fibrotic areas especially in the peritubular region without affecting the glomeruli (Figure 1(a), lower panel). The histological phenotype was increased in the HPD7 mice as compared to the HPD4 mice.

To quantify kidney function, we performed FITC-inulin clearance, which showed decreased GFR in both HPD groups as compared to the control SCD group, but significance was only reached in the HPD7 group compared to SCD mice (Figures 1(b) and 1(c)). In line, serum urea was increased in both HPD groups compared to SCD (Figure 1(d)).

**Figure 2:**
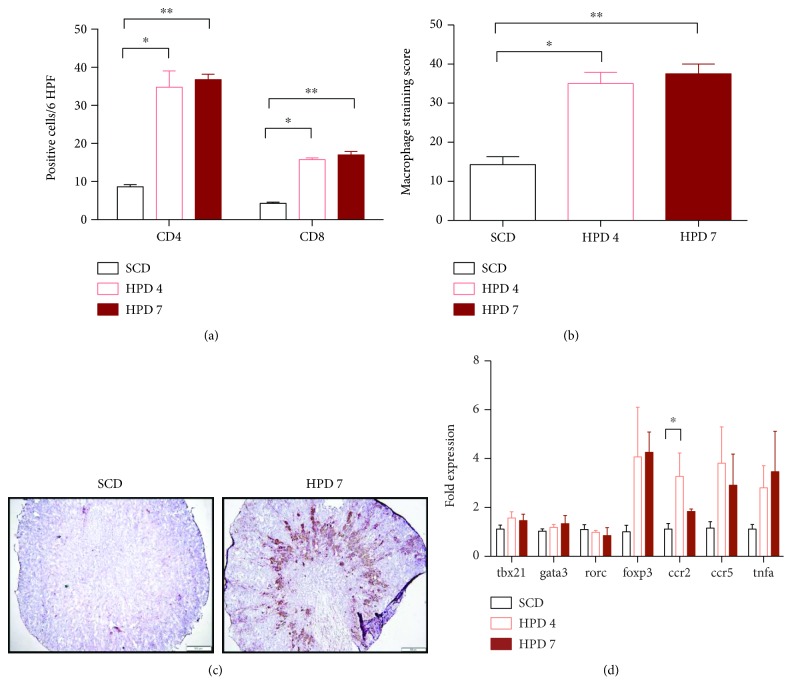
Immune cell infiltration into CKD kidneys. Kidneys of mice (SCD: *n* = 8; HPD4: *n* = 4; and HPD7: *n* = 4) were stained for (a) CD4^+^ and CD8^+^ T cells or (b) CD68^+^ macrophages. (c) Representative pictures of CD68 stained kidney tissue from SCD and HPD7 mice are shown. (d) Quantitative PCR of respective genes was performed in kidney tissue. The fold expression compared to the mean mRNA expression of SCD mice is provided. ^∗^*p* < 0.05 and ^∗∗^*p* < 0.01.

The tubular calcium hydroxyapatite precipitations were associated with a prominent renal infiltration of leukocytes (Figure 2). CD4^+^ and CD8^+^ T cells were found to infiltrate the kidneys of mice treated with HPD throughout the interstitial area, whereas significantly decreased numbers were detected in the SCD group (Figure 2(a)). The infiltrate mainly consisted of CD68^+^ macrophages, which were found in significantly increased numbers in HPD-treated mice (Figures 2(b) and 2(c)). In line, we detected an increase in macrophage mRNA markers such as *C-C chemokine receptor* (*Ccr*) *2* and *Ccr 5* as well as *Tnfa* in the kidneys of HPD mice (Figure 2(d)). The TH1, TH2, and TH17 markers *Tbx21*, *Gata3*, and *Rorc*, respectively, were not regulated on the transcriptional level (Figure 2(d)). Only the regulatory T cell marker *FoxP3* was increased in HDP mice compared to SCD-treated mice (Figure 2(d)).

### 3.2. Cardiovascular Changes in Mice Treated with High-Phosphate Diet for 7 Days Followed by 84 Days Standard Chow

**Figure 3:**
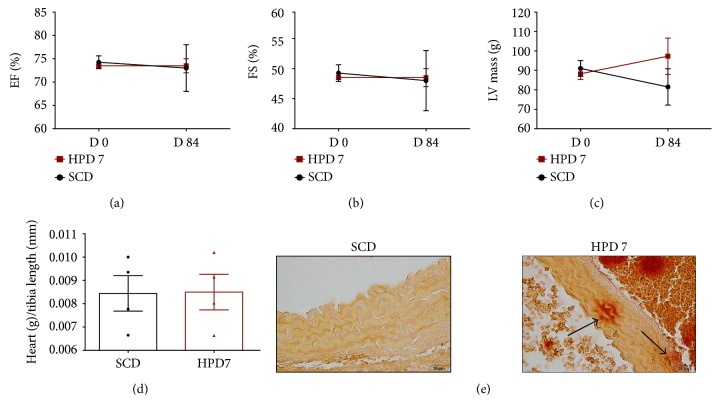
Cardiovascular phenotype of the CKD model. Echocardiography was performed before starting the CKD model (*n* = 4 per group). One group of mice was fed with HPD for 7 days (HPD7) and followed until day 84. This group was compared to mice fed with SCD throughout the study period. Both groups were evaluated by echocardiography on day 84 (SCD: *n* = 4; HPD7: *n* = 2). Evaluation included (a) EF, (b) FS, and (c) LV mass. (d) Abdominal aortas of the 7-day HPD group and the SCD group were stained with alizarin red. Calcifications were detected in the media of aortas (arrow). Representative pictures are shown.

HPD7 mice or SCD mice were evaluated by echocardiography before starting the diet and at day 84 of follow-up. A major limitation of the echocardiographic observations is the fact that (*n*) numbers on day 84 in HPD7 mice were small (*n* = 2). No difference in left ventricular (LV) systolic function (EF and FS) or mass was found between the mice at baseline. At day 84, preliminary observations in HPD7 mice showed no difference in EF and SF compared to SCD mice, while LV mass tended to increase in HPD7 mice (Figures 3(a), 3(b), and 3(c)). Heart weights did not differ between HPD7 and SCD mice (Figure 3(d)). Of note, no significant myocardial calcifications were detected in our mice (data not shown).

The abdominal aortas of mice were evaluated for calcification by performing alizarin stains. Calcified areas were detected in the media of the aortas of HPD7 mice, whereas no calcifications were found in SCD mice (Figure 3(e)).

### 3.3. A Model of CKD-Associated Low-Turnover Bone Disease

**Figure 4:**
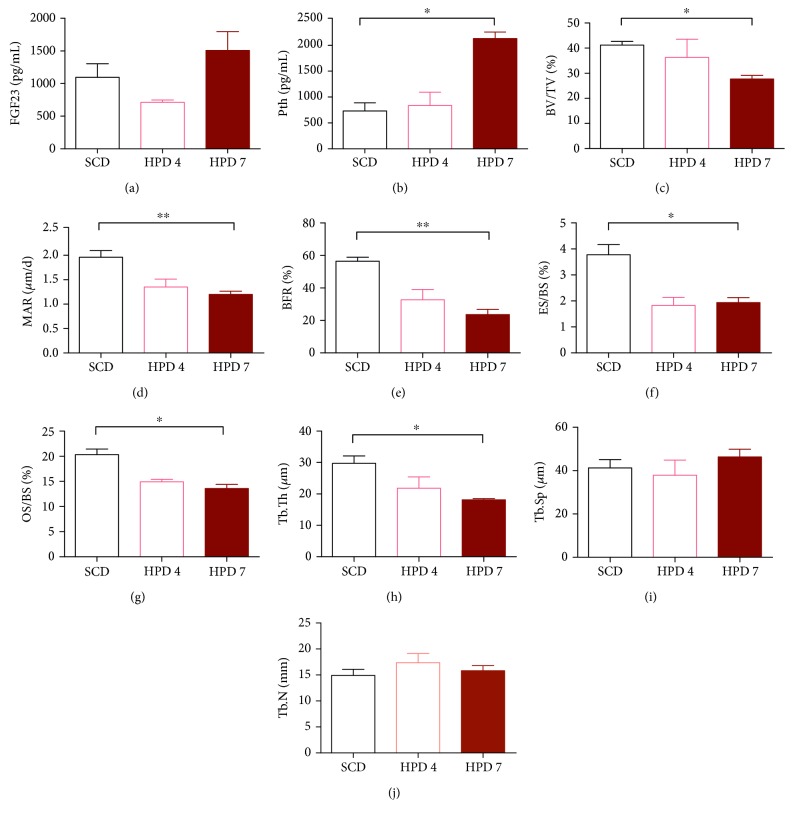
Bone histomorphometry and histology of CKD mice. Mice were either fed with HPD for 4 (HPD4, *n* = 4) or 7 days (HPD7, *n* = 4) and then followed on SCD until day 84. They were compared with mice on SCD for the complete study period (SCD, *n* = 8). On day 84, (a) serum Pth levels and (b) FGF23 levels were evaluated. Furthermore, mice were calcein-labelled and the tibia was analysed by using bone histomorphometry. (c) Bone volume/tissue volume (BV/TV), (d) mineral apposition rate (MAR), (e) bone formation rate (BFR), (f) eroded surface/bone surface (ES/BS), (g) osteoid surface/bone surface (OS/BS), (h) trabecular thickness (Tb.Th), (i) trabecular separation (Tb. Sp.), and (j) trabecular number (Tb.N) are provided. ^∗^*p* < 0.05 and ^∗∗^*p* < 0.01.

The surrogate parameters for bone disease in CKD parathyroid hormone (Pth) and fibroblast growth factor 23 (FGF23) were evaluated. Whereas we detected a significant increase in Pth in mice on day 84 in the HPD7 group compared to SCD mice, no difference was found in FGF23 levels between the three groups (Figures 4(a) and 4(b)).

Mice were calcein-labelled and after sacrifice, tibias were evaluated on day 84 by bone histomorphometry. Overall, the bone structure was more deteriorated in the HPD7 group. In detail, we detected a decrease in the bone volume/tissue volume (BV/TV), mineral apposition rate (MAR), bone formation rate (BFR), eroded surface/bone surface (ES/BS), osteoid surface/bone surface (OS/BS), and trabecular thickness (Tb.Th) in the HPD4 and HPD7 group as compared to SCD mice, but significance was only reached in the HPD7 group (Figures 4(c), 4(d), 4(e), 4(f), 4(g), and 4(h)). The trabecular separation (Tb.Sp) and trabecular number (Tb.N) did not differ between the groups (Figures 4(i) and 4(j)).

## 4. Discussion

In this manuscript, we present a new model of CKD, which has features of MBD as shown by arterial media calcification, secondary hyperparathyroidism, and low-turnover bone disease.

The classical CKD model is the 5/6 nephrectomy model, which is difficult to perform consistently in mice, since robust CKD is in most cases not induced. Thus, nowadays, most groups use the CKD model established by Gagnon et al., where the majority of the renal surface of one kidney is coagulated followed by nephrectomy of the other kidney after some weeks of recovery [18]. Nevertheless, mortality during and after this procedure is high and surgical procedures are necessary to induce CKD [13], which is an inherent source of bias. In our model, we do not need surgical interventions and the mortality of these mice is very low (5 to 10%; data not shown) since we stop HPD on day 4 or 7, respectively. As shown previously, mortality increases rapidly in our mice when fed with HPD for more than 10 days [19]. Of note, choosing the DBA/2 strain is of critical importance to induce CKD, since C57BL/6 mice do not develop critical calcification neither in the kidney nor in the cardiovascular system [20]. This is explained by the fact that DBA/2 mice have an alternative splice variant of the *Abcc6* gene resulting in an increased susceptibility to develop tissue calcification [14–16].

In our CKD model, mice suffer from CKD reflecting CKD stage 3 in humans since GFR declined by 50% compared to SCD mice, which differs to the surgically induced CKD stage 5 model [13, 21, 22]. Thus, our model provides the opportunity to study early CKD-MBD changes in different organs, which is of particular interest since only early interventions seem to improve mortality and morbidity in our CKD patients.

Our CKD mice develop cardiovascular changes such as media sclerosis in the abdominal aorta. From our data, we cannot clearly tell, whether the mice also develop concentric left ventricular hypertrophy since we have low (*n*) numbers in echocardiography and heart weights did not differ between the groups. In HPD-induced acute kidney injury model due to phosphate nephropathy, we found the picture of dystrophic cardiac calcinosis resulting in the significantly increased mortality in the mice [19]. In the presented CKD model, the cardiac picture looked differently since we did not detect relevant calcifications in the myocardium (data not shown), but preliminary observations by echocardiography showed that the mice developed some extent of cardiac hypertrophy probably due to hypertension which more closely resembles the human CKD-MBD phenotype. Nevertheless, increasing (*n*) numbers in echocardiography and further evaluations such as blood pressure measurement are necessary to characterize the cardiac phenotype in detail. In the presented CKD model, we detected media sclerosis predominantly in the abdominal aorta, which is in line with our previous data published in an HPD-induced acute kidney injury mouse model [23]. Both in CKD patients and in our CKD mouse model, there is a different susceptibility of the ascending aorta and the abdominal aorta to vascular media calcification [23]. The mechanisms for this observational finding remain elusive so far. It was speculated that due to their different embryonic origin, vascular smooth muscle cells in different parts of the aorta have a different susceptibility to calcification [23, 24].

In our CKD mice, we detected a low-turnover bone disease by performing bone histomorphometry even though Pth levels were significantly increased in HPD mice. This highlights the fact that Pth is an imperfect marker for evaluating bone disease in CKD, and bone histomorphometry should be considered as gold standard for diagnosing bone disease in CKD [8, 9]. Interestingly, we detected FGF23 levels to be increased only after 14 days (data not shown), whereas no difference was detectable on day 84. Obviously, the early FGF23 increase is a physiological reaction to the HPD in order to clear the excessive oral phosphate intake but normalizes after the long observation interval of 11 weeks on standard chow diet. Nevertheless, it would be interesting to also study FGF23 expression in the bone of our mice since increased expression levels have been described recently in the bone of CKD patients with renal osteodystrophy [25]. To our knowledge, this is the first experimental CKD model describing a low-turnover bone disease. Others published a model of renal osteodystrophy in a surgically induced subtotal nephrectomy/CKD model [13, 21, 22]. Contrary to our model, histomorphometry was evaluated in the lumbar vertebrae rather than in the tibiae [13]. They performed microCT in the tibia-trabecular region and found increased bone volume and decreased mineral density in the metaphysis of their mice [13], whereas we found decreased bone volume and mineralization in the tibia of our CKD model. Nevertheless, we need to further analyse the bone disease in our model by methods of histology and confocal microscopy to gain additional insights into our newly described mouse model.

In summary, our mouse model offers a new and easy to perform reproducible tool to study the pathogenesis and treatment options of CKD-MBD and especially of low-turnover bone disease in CKD.
